# Supplementary material for: Evaluation of leukopenia during sepsis as a marker of sepsis-defining organ dysfunction
Source: PLoS One. 2021 Jun 24;16(6):e0252206. doi: 10.1371/journal.pone.0252206 (PMC8224900; doi:10.1371/journal.pone.0252206)
Supplement: S1 Table — (DOCX) [file pone.0252206.s001.docx]

**Supplemental Table 1 – Odds Ratios for Neutropenia Models**

| Covariate | OR (C.I.) | p-value |
| --- | --- | --- |
| Neutropenia (model 1 -unadjusted) | 1.9 (1.4-2.7) | <0.001 |
| Normal neutrophils | 0.8 (0.7 – 0.9) | 0.001 |
| Neutrocytosis *(reference)* | 1 | - |
| Neutropenia (model 2 - Sepsis-3 baseline model covariates) | 2.3 (1.6 – 3.2) | <0.001 |
| Normal neutrophils | 0.8 (0.7 – 0.9) | 0.003 |
| Neutrocytosis *(reference)* | 1 | - |
| Neutropenia (model 3 - Sepsis-3 baseline model and maximum SOFA score covariates) | 1.6 (1.1-2.3) | 0.01 |
| Normal neutrophils | 0.8 (0.6-0.9) | 0.001 |
| Neutrocytosis *(reference)* | 1 | - |
| Neutropenia (model 4 –maximum SOFA score covariates) | 1.4 (1.0-1.9) | 0.09 |
| Normal neutrophils | 0.7 (0.6 -0.9) | <0.001 |
| Neutrocytosis *(reference)* | 1 | - |

Covariates included in each model included:

Model 1: WBC category

Model 2: WBC category, age (fractional polynomial), race, gender, co-morbidity index (fractional polynomial)

Model 3: WBC category, age(fractional polynomial), race, gender, SOFA score, co-morbidity index (fractional polynomial)

Model 4: WBC category, SOFA score

*Definition of abbreviation:* SOFA = sequential organ function assessement; SD = standard deviation; WBC = white blood cell count; OR = Odds Ratio; CI = Confidence Interval.
