# Supplementary material for: Evaluation of leukopenia during sepsis as a marker of sepsis-defining organ dysfunction
Source: PLoS One. 2021 Jun 24;16(6):e0252206. doi: 10.1371/journal.pone.0252206 (PMC8224900; doi:10.1371/journal.pone.0252206)
Supplement: S2 Table — (DOCX) [file pone.0252206.s002.docx]

**Supplemental Table 2. Odds Ratios for Lymphopenia models**

| Covariate | OR (C.I.) | p-value |
| --- | --- | --- |
| Lymphopenia (model 1 -unadjusted) | 1.1 (0.8-1.5) | 0.66 |
| Normal lymphocytes | 0.9 (0.6 – 1.2) | 0.43 |
| lymphocytosis *(reference)* | 1 | - |
| Lymphopenia (model 2 - Sepsis-3 baseline model covariates) | 1.0 (0.7 – 1.3) | 0.82 |
| Normal lymphocytes | 0.8 (0.6 – 1.1) | 0.24 |
| lymphocytosis *(reference)* | 1 | - |
| Lymphopenia (model 3 - Sepsis-3 baseline model and maximum SOFA score covariates) | 1.0 (0.7-1.5) | 0.80 |
| Normal lymphocytes | 1.0 (0.7-1.3) | 0.83 |
| lymphocytosis *(reference)* | 1 | - |
| Lymphopenia (model 4 –maximum SOFA score covariates) | 1.1 (0.8-1.6) | 0.46 |
| Normal lymphocytes | 1.0 (0.7 -1.4) | 0.96 |
| lymphocytosis *(reference)* | 1 | - |

Covariates included in each model included:

Model 1: WBC category

Model 2: WBC category, age (fractional polynomial), race, gender, co-morbidity index (fractional polynomial)

Model 3: WBC category, age(fractional polynomial), race, gender, SOFA score, co-morbidity index (fractional polynomial)

Model 4: WBC category, SOFA score

*Definition of abbreviation:* SOFA = sequential organ function assessement; SD = standard deviation; WBC = white blood cell count; OR = Odds Ratio; CI = Confidence Interval.
